# Supplementary figures and images for: Orange-spotted grouper nervous necrosis virus-encoded protein A induces interferon expression via RIG-I/MDA5-MAVS-TBK1-IRF3 signaling in fish cells
Source: Microbiol Spectr. 2023 Dec 14;12(1):e04532-22. doi: 10.1128/spectrum.04532-22 (PMC10783131; doi:10.1128/spectrum.04532-22)

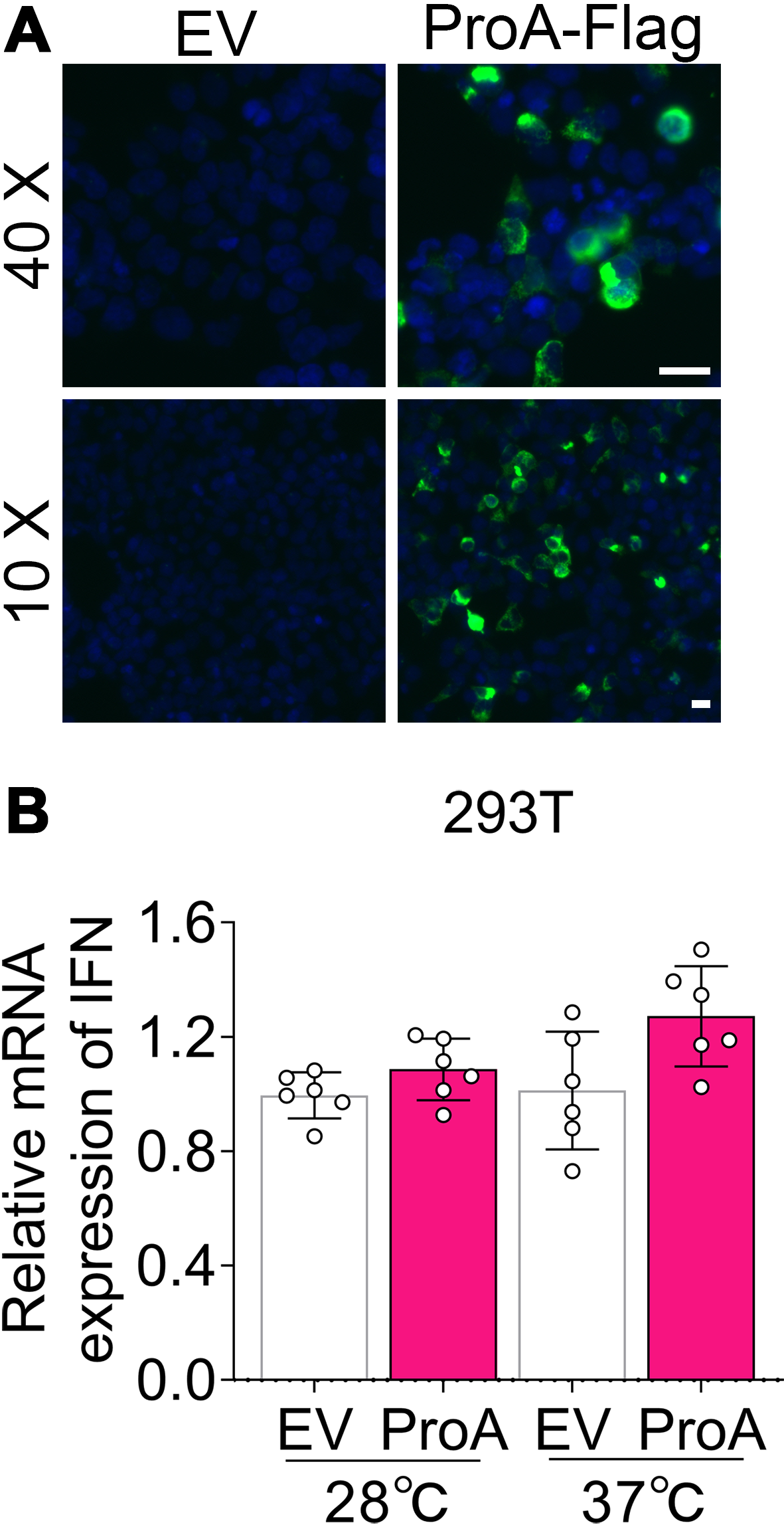

Supplement: Fig. S1 — Overexpression of ProA in 293T cells cannot induce human IFN expression. [file spectrum.04532-22-s0001.tif]

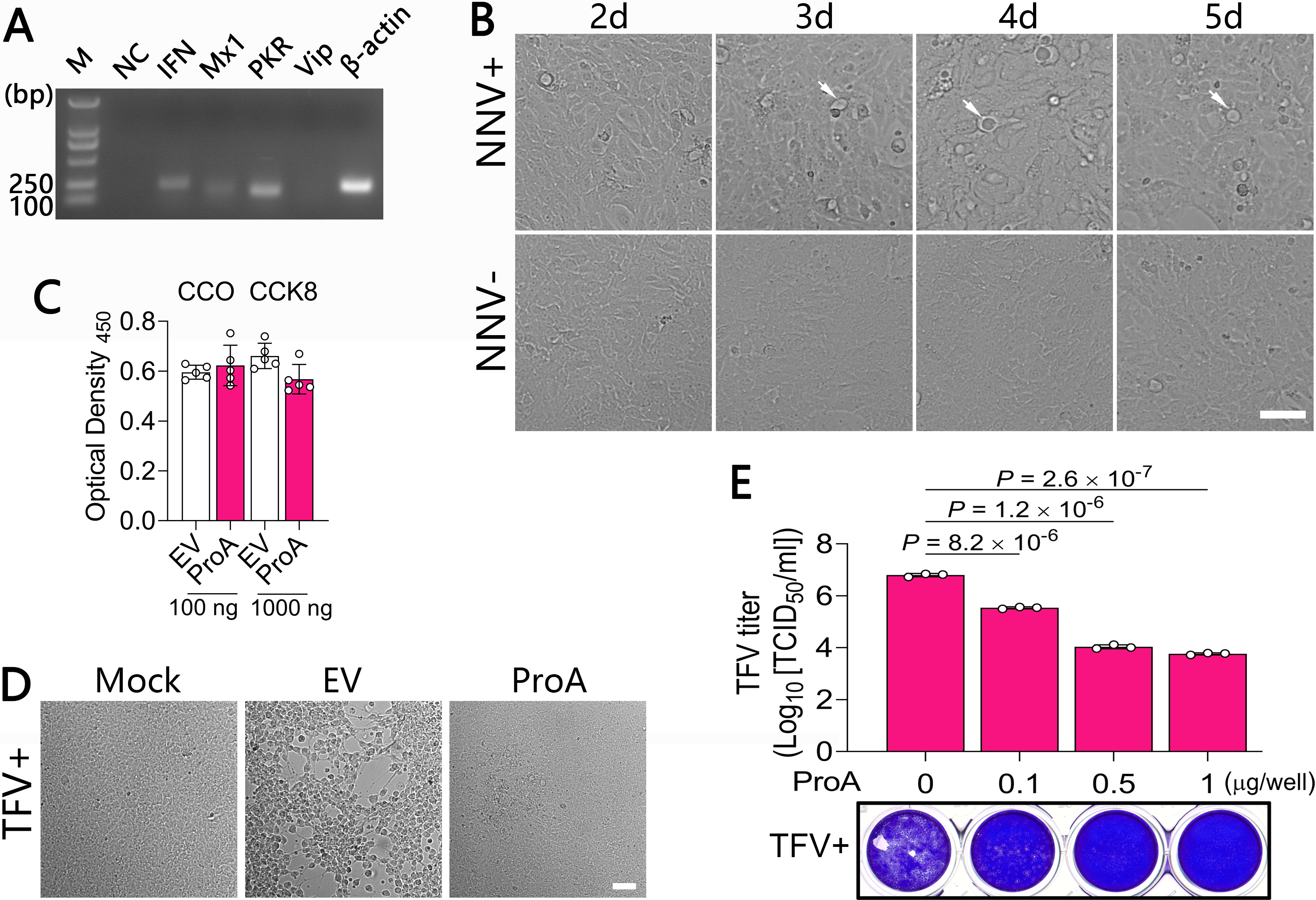

Supplement: Fig. S2 — CCO is an ideal cell line for the study of ProA-mediated IFN activation. [file spectrum.04532-22-s0002.tif]

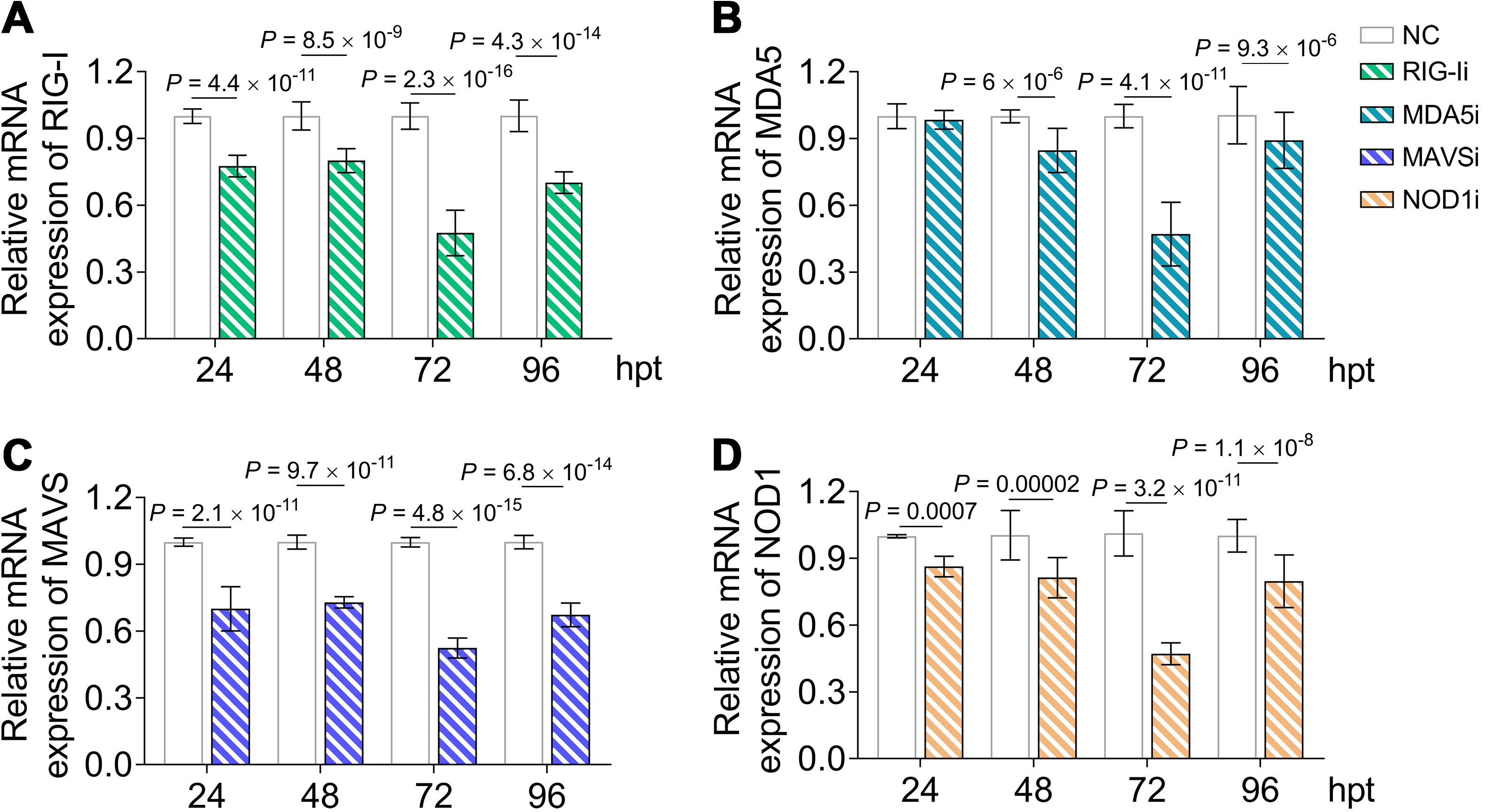

Supplement: Fig. S3 — RNAi efficiency in FHM cells. [file spectrum.04532-22-s0003.tif]

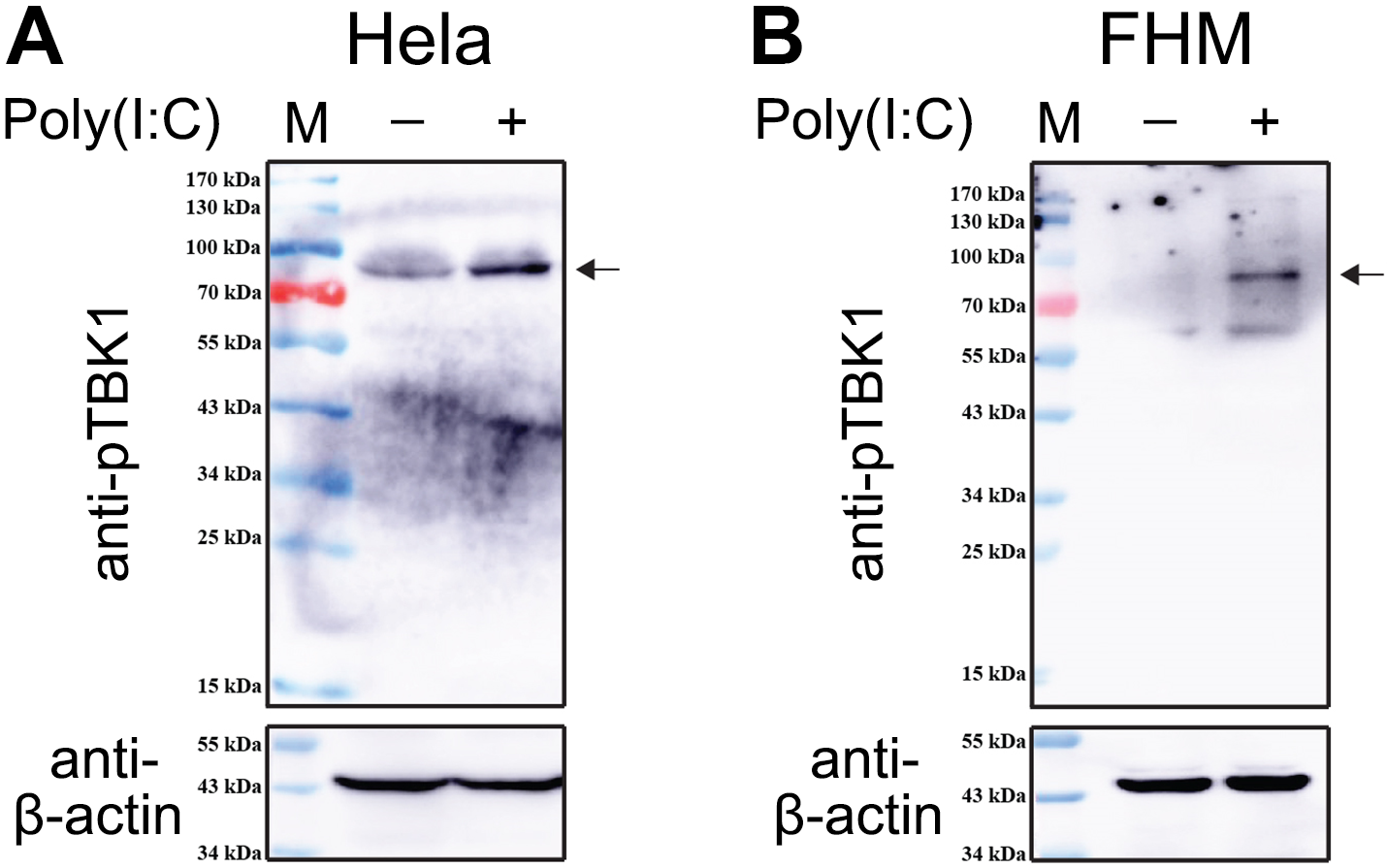

Supplement: Fig. S4 — FHM pTBK1 could be detected by the commercial antibody. [file spectrum.04532-22-s0004.tif]
