# Supplementary material for: Orange-spotted grouper nervous necrosis virus-encoded protein A induces interferon expression via RIG-I/MDA5-MAVS-TBK1-IRF3 signaling in fish cells
Source: Microbiol Spectr. 2023 Dec 14;12(1):e04532-22. doi: 10.1128/spectrum.04532-22 (PMC10783131; doi:10.1128/spectrum.04532-22)
Supplement: Supplemental legends — Legends for Fig. S1 to S4. [file spectrum.04532-22-s0005.docx]

Orange-spotted grouper nervous necrosis virus-encoded Protein A induces interferon expression via RIG-I/MDA5-MAVS-TBK1-IRF3 signaling in fish cells

Siyou Huang^a,b,c,d^, Yi Huang^a,b,c,d^, Taowen Su^a,b,c,d^, Runqing Huang^e^, Lianpan Su^a,b,c,d^, Yujia Wu^a,b,c,d^, Shaoping Weng^a,b,c,d^, Jianguo He^a,b,c,d^#, Junfeng Xie^a,b,c,d^#

^a^ State Key Laboratory of Biocontrol, School of Life Sciences, Sun Yat-sen University, Guangzhou, People’s Republic of China

^b^ Southern Marine Science and Engineering Guangdong Laboratory (Zhuhai), Zhuhai, People’s Republic of China

^c^ China-ASEAN Belt and Road Joint Laboratory on Marine Aquaculture Technology, School of Life Sciences, Sun Yat-sen University, Guangzhou, People’s Republic of China

^d^ Institute of Aquatic Economic Animals and Guangdong Provincial Key Laboratory of Improved Variety Reproduction in Aquatic Economic Animals, School of Life Sciences, Sun Yat-sen University, Guangzhou, People’s Republic of China

^e^ School of Life Science, Huizhou University, Huizhou, People’s Republic of China

**Supplemental materials – Figure legend**

**Fig. S1** Overexpression of ProA in 293T cells cannot induce human IFN expression. A. IFA detection of flag-tagged ProA overexpression in 293T cells. The plasmid of ProA-Flag and empty vector (EV) was transfected into 293T and ProA-Flag was detected by anti-Flag and Alexa fluor 488 (green) antibodies by IFA. Magnifications of 10× and 40× were shown and white bars indicated 50 μm. B. IFN mRNA detection in ProA- and EV-transfected 293T in different culture temperatures. The 293T cells were transfected with the plasmids of ProA or EV and cultured at 28 ℃ or 37 ℃ for 48 h respectively. The mRNA level of IFN was determined by RT-PCR. For B, *n* = 6 independent experiments. Means ± s.d. are shown, and *P* values were calculated using two-tailed unpaired Student’s *t*-test.

**Fig. S2** CCO is an ideal cell line for the study of ProA-mediated IFN activation. A. The cDNA fragments of IFN, Mx1, Viperin (Vip), PKR, and β-actin could be detected in untreated CCO cells by RT-PCR. B. CCO cells were permissive to NNV. The monolayer of CCO cells was infected with OGNNV (MOI=10). Typical cytopathic effects (CPE) of cell enlargement and vacuolization (white arrows) were observed after 3 d post-infection and no complete cell destruction was observed at 5 d post-infection. C. Overexpression of ProA showed no cytotoxicity to CCO cells. The overdose of the plasmid of ProA and EV was transfected and 48 h later CCO cells were collected for CCK-8 assay to detect cell viability. D. TFV replication was inhibited by ProA expression. TFV infection (MOI=2) was performed 48 h after ProA and EV were transfected into CCO cells. CPE was observed at 48 h post-infection. E. Different doses of ProA were transfected and TFV infection was performed 48 h post-transfection. Forty-eight hours post-infection, monolayers of CCO cells were stained with crystal violet and the virus titer of the supernatants was determined. For C and E, experiments were repeated at least three times independently. Means ± s.d. are shown, and *P* values were calculated using two-tailed unpaired Student’s *t*-test. For B and D, white bars indicated 50 μm.

**Fig. S3** RNAi efficiency in FHM cells. The mRNA level of RIG-I (A), MDA5 (B), MAVS (C), and NOD1 (D) was analyzed by RT-qPCR at different time points post-transfection of the corresponding siRNAs. The highest knockdown efficiency was about 50% at 72 hpt.

**Fig. S4** FHM phosphorylated TBK1 (pTBK1) could be detected by the commercial antibody. Immunoblot analysis of pTBK1 was performed to verify the applicability of a commercial antibody (5483S, Cell Signaling) on Hela (A) and FHM (B) cells transfected with poly(I:C) (+) or not (-). The internal control for this immunoblot analysis was set as β-actin. The band size of the protein marker (M) was shown on the left. The arrow indicated the band of phosphorylated TBK1.
